# Bevacizumab improves tumor infiltration of mature dendritic cells and effector T-cells in triple-negative breast cancer patients

**DOI:** 10.1038/s41698-021-00197-w

**Published:** 2021-06-29

**Authors:** Yves Boucher, Ashwin S. Kumar, Jessica M. Posada, Evisa Gjini, Kathleen Pfaff, Mikel Lipschitz, Ana Lako, Dan G. Duda, Scott J. Rodig, F. Stephen Hodi, Rakesh K. Jain

**Affiliations:** 1 Edwin L. Steele Laboratories, Department of Radiation Oncology Massachusetts General Hospital and Harvard Medical School, Boston, MA USA; 2 Harvard–MIT Division of Health Sciences and Technology, Massachusetts Institute of Technology, Cambridge, MA USA; 3 Department of Pathology, Brigham and Women’s Hospital, Boston, MA USA; 4 Center for Immuno-oncology, Dana-Farber Cancer Institute, Boston, MA USA; 5 Bristol Myers Squibb, Cambridge, MA USA; 6 Department of Medical Oncology, Dana-Farber Cancer Institute, Boston, MA USA

**Keywords:** Tumour immunology, Breast cancer

## Abstract

A single dose of bevacizumab reduced the density of angiopoietin-2-positive vessels while improving the infiltration of CD4+ T and CD8+ T cells, and mature dendritic cells in patients with primary triple-negative breast cancer. Our findings provide a rationale for including bevacizumab during neoadjuvant treatment to enhance the efficacy of immune checkpoint blockers in this disease.

Approximately 15–20% of all breast cancers lack expression of HER2 and hormone receptors and are referred to as triple-negative breast cancer (TNBC). A randomized phase III trial demonstrated significant efficacy of neoadjuvant anti-PD-1 antibody pembrolizumab combined with chemotherapy for localized TNBC^1^. However, response was seen in less than 30% of patients. In addition, in metastatic TNBC, pembrolizumab and chemotherapy increased median progression-free survival by 2–4 months, more often in PD-L1-positive tumors^2^. Therefore, new approaches are needed to further improve the efficacy of immunotherapy for TNBC.

Using orthotopic breast cancer models in mice, we previously found that blocking vascular endothelial growth factor receptor-2 (VEGFR2) normalizes the tumor vasculature, polarizes immunosuppressive tumor-associated macrophages (TAMs) to an immunostimulatory phenotype, enhances the infiltration of CD8+ T cells, and improves the effectiveness of a cancer vaccine^3^. We also found that anti-VEGFR2 therapy decreases angiopoietin-2 (Ang2) expression in a breast cancer model^4^. Ang2 can destabilize blood vessels, increase the recruitment of immunosuppressive cells, and is associated with immunotherapy resistance in melanoma patients^5,6^. Thus, reduction in Ang2 expression may contribute to extending vascular normalization and improving anti-tumor immune responses^7^. Additionally, VEGF can directly inhibit the maturation of dendritic cells (DCs)^8^.

We hypothesized that the anti-VEGF antibody bevacizumab could normalize tumor vessels, reduce Ang2 levels, and increase the infiltration by T cells and other immunostimulatory cells, including DCs, in human TNBC. To this end, we assessed the effects of VEGF blockade on the vasculature and intratumoral infiltration by immune cells in 10 paired-biopsies prior to treatment and 2 weeks after a single dose of bevacizumab in a phase II trial of neoadjuvant bevacizumab (10 mg/kg) followed by bevacizumab combined with dose-dense chemotherapy in TNBC patients^9^ (see Methods). We previously reported that bevacizumab decreased circulating Ang2 levels and induced vascular normalization in patients with a sufficiently high tumor microvascular density at baseline^9^. Here, we used multiplex immunofluorescence to quantify the density of CD31+Ang2+ and CD31+Ang2− blood vessels and density of T cells, TAMs, and DCs in these biopsies.

Bevacizumab significantly reduced total CD31+ and CD31+Ang2+ but not CD31+Ang2− vessel density (Fig. 1a–d), consistent with vascular normalization. As seen in preclinical models^3^, VEGF blockade significantly increased the overall infiltration by CD8+ T cells, including CD8+PD-1+, CD8+PD-1−, and CD8+granzyme-B+ (GzmB+) T-cell subsets in TNBC (Fig. 2a–e, Supplementary Fig. 1). Moreover, we found a non-significant trend for increased density of CD8+ T cells post-bevacizumab in lesions with fewer CD31+Ang2+ vessels (Supplementary Fig. 1). The fraction of CD8+PD-1+ T cells, whose increase was recently shown to associate with improved survival in TNBC^10^, did not change post-bevacizumab (Supplementary Fig. 1). PD-1^hi^ phenotype in CD8+ T cells in TNBC is a marker of T-cell exhaustion but is also associated with biomarkers of activation (i.e., IFNγ+, GzmB+) more than PD-1− or PD-1^lo^ phenotype^10^.

**Fig. 1:**
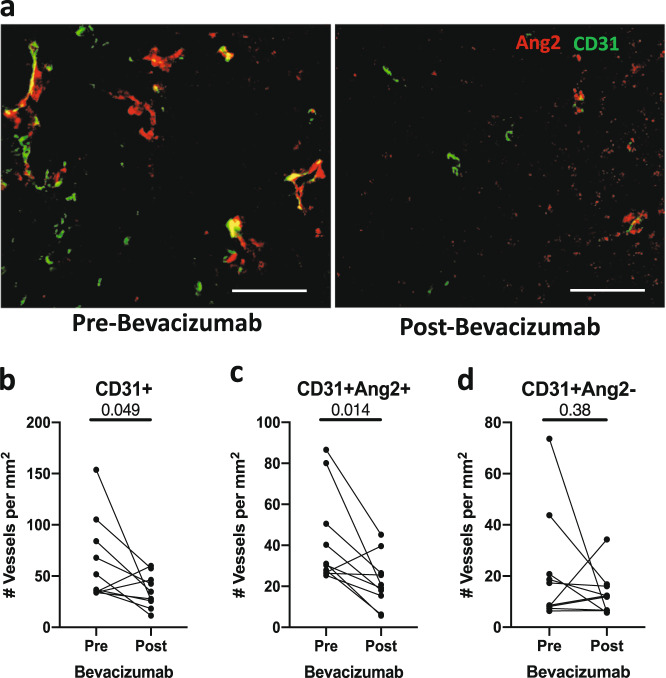
Bevacizumab reduced the CD31+Ang2+ vessel density. **a** Representative immunofluorescence for CD31 and Ang2 pre- and post-bevacizumab (images from paired samples of same patient); scale bar = 50 µm. Quantitative analyses of TNBC vessels (**b**–**d**).

**Fig. 2:**
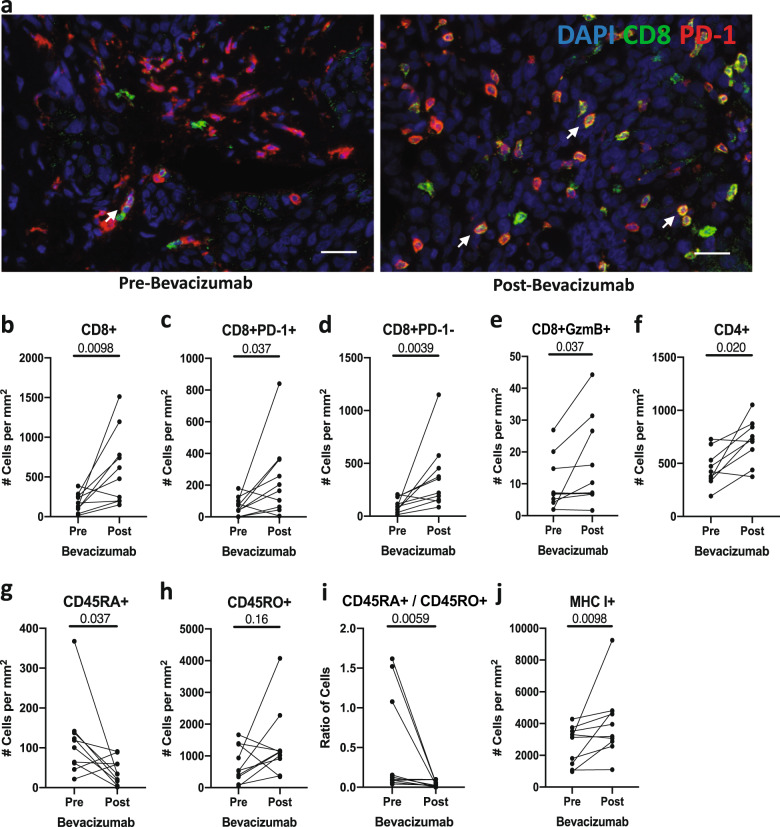
Bevacizumab increased the CD8+ and CD4+ T-cell density and MHC-I expression. **a** Representative multiplex immunofluorescence for CD8 and PD-1 pre- and post-bevacizumab (images from paired samples of the same patient). CD8+PD-1+ T cells (white arrows); scale bar = 50 µm. **b**–**f** Quantitative analyses of overall CD8+ (**b**), CD8+PD-1+ (**c**), CD8+PD-1− (**d**), CD8+GzmB+ (**e**), and CD4+ T cells (**f**). **g**–**j** Quantitative analyses of CD45RA+ (**g**) and CD45RO+ (**h**) T-cells, CD45RA+/CD45RO+ ratio (**i**), and MHC-I+ cells (**j**).

Our analysis further revealed that bevacizumab significantly increased the intratumoral density of CD4+ T cells but not CD4+FOXP3+ T cells (Fig. 2f, Supplementary Fig. 1). Interestingly, we found a significant inverse correlation between CD4+ T cell and CD31+Ang2+ vessel density post-bevacizumab (Supplementary Fig. 1). CD4+ T cells mediate vascular normalization in breast cancer models^11^, and bevacizumab-induced vascular normalization in these TNBC patients^9^ via Ang2 suppression could mediate the increase in CD8+ T-cell density. Indeed, dual VEGF/Ang2 blockade increased intratumoral density of CD8+ T cells in murine tumors^7^.

We also determined the effect of VEGF blockade on naïve (CD45RA+) and memory (CD45RO+) T cells, and MHC-I expression. Bevacizumab significantly reduced CD45RA+ T-cell density (Fig. 2g) and tended to increase CD45RO+ memory T-cell density in 7/10 paired-biopsies (Fig. 2h), resulting in a significant decrease in CD45RA+/CD45RO+ T-cell ratio (Fig. 2i). Moreover, bevacizumab significantly increased the expression of MHC-I (Fig. 2j), in line with findings in renal cell carcinoma^12^. Hence, bevacizumab can promote the maturation of memory T cells and MHC-I expression in TNBC.

Moreover, bevacizumab treatment induced a significant increase in both CD11c+CD163−CD68− and CD11c+CD163+CD68− DC density in TNBC tissues (Fig. 3a–c). Bevacizumab also induced a significant increase in density of CD163+CD11c−CD68− cells (Fig. 3d) but not CD163+CD68+CD11c− (M2-like) TAMs (Fig. 3e) or CD68+CD163−CD11c− or CD11c+CD68+CD163− (M1-like) TAMs (Supplementary Fig. 1). Bevacizumab-induced infiltration of CD11c+CD163+CD68– DCs in TNBC was correlated with infiltration of CD8+ (Rho = 0.67) and CD8+PD-1+ T-cells (Rho = 0.81), and MHC-I expression (Rho = 0.81) (Supplementary Fig. 2). CD163 is a biomarker of M2-like TAMs and the mature inflammatory DC3 subset^13^. DC3s activate naïve T cells and promote the recruitment of memory T cells in breast cancer^13^. Our results suggests that inhibition of VEGF—a known inhibitor of DC activity^8^—can enhance the maturation of DCs in TNBC.

**Fig. 3:**
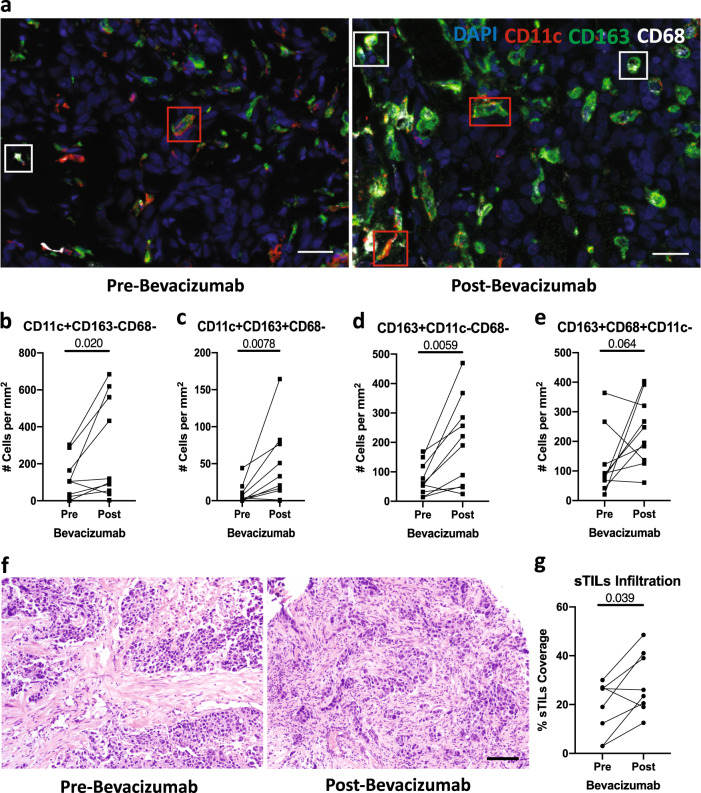
Bevacizumab increased CD11c+CD163−CD68− and CD11c+CD163+CD68− DC density. **a** Representative multiplex immunofluorescence for CD11c, CD163, and CD68 in TNBC pre- and post-bevacizumab (images from paired samples of the same patient); scale bar = 50 μm. Red boxes identify CD11c+CD163+CD68− DCs, white boxes identify CD163^+^CD68^+^CD11c− TAMs. **b**–**e** Quantitative analyses of CD11c+CD163−CD68− (**b**), CD11c+CD163+CD68− (**c**), CD163+CD11c−CD68− (**d**), and CD163+CD68+CD11c− (**e**) cells. **f** H&E image of sTILS pre- and post-bevacizumab (images from paired samples of the same patient); scale bar = 100 µm. **g** Quantitative analysis of sTILs.

Finally, bevacizumab significantly increased the percentage of stromal tumor-infiltrating lymphocytes (sTILs), a well-established prognostic marker in TNBC (Fig. 3f–g)^14^. The percentage of sTILs also correlated with MHC-I expression pre- (Rho = 0.95) and post-bevacizumab (Rho = 0.83) (Supplementary Fig. 2).

The post-bevacizumab bimodal response of several immune phenotypes (e.g., CD8+ T cells, CD11c+CD163+CD68− DCs) suggested a differential response between patients. We used the unbiased K-means clustering algorithm to analyze data. K-means clustering separated patients into 2 clusters pre- and post-bevacizumab (Supplementary Fig. 3). In cluster 1 bevacizumab-induced enhancement of CD8+ T cells, CD8+PD-1+ T cells, CD11c+CD163+CD68− DCs and CD163+CD11c−CD68− was at a maximum and associated with higher infiltration of CD11c+CD163+CD68− and CD163+CD11c−CD68− cells pre-bevacizumab, as well as lower densities of CD31+Ang2+ vessels pre-bevacizumab. In cluster 2 the changes for the same four immune biomarkers were relatively small (Supplementary Fig. 3).

Our study has several limitations. A single biopsy may not reflect the heterogeneity of immune microenvironment. Also, the biopsy procedure may produce a focal inflammatory response. These limitations notwithstanding, our results show that bevacizumab can increase intratumoral infiltration by sTILs, CD4+ T cells, CD8+ (including PD-1+) T cells, CD8+GzmB+ T cells, and CD11c+CD163+CD68− DCs in primary TNBC. The effects of bevacizumab treatment on DCs and T cells suggest that VEGF blockade could enhance the efficacy of immunotherapy in TNBC. Indeed, clinical findings strongly suggest that improving the infiltration of sTILs and CD8+ T cells can improve the efficacy of immunotherapy in TNBC^14,15^. The changes in infiltrating immune cells induced by bevacizumab should be further evaluated in clinical studies as predictive dynamic biomarkers of treatment efficacy and patient selection for combinations with immunotherapy. This is particularly critical for TNBC, since the vast majority of patients do not respond to immunotherapy. Our findings, along with proven efficacy of combined anti-PD-1/PD-L1 and anti-VEGF/R agents in multiple malignancies, provide a strong rationale for this approach in neoadjuvant setting in TNBC patients.

## Methods

### Ethics of study design and consent

This study was approved by the Dana–Farber/Harvard Cancer Center Institutional Review Board. Written informed consent was required for enrollment. The trial is registered at ClinicalTrials.gov (NCT00546156).

### Patient characteristics

Enrollment required a pathological diagnosis of adenocarcinoma of the breast. Eligible TNBC patients were negative for ER, PR, and HER2, had a breast lesion ≥1.5 cm, and no evidence of distal metastasis. Patients with bilateral cancer were eligible as long as one cancer was eligible. Patients also required sufficient hematopoietic, hepatic, and renal function, along with a left ventricular ejection fraction ≥50%. Patients with any HER2-positive disease (amplified by FISH or IHC), a history of prior myocardial infarction, uncontrolled hypertension, ≥grade 2 neuropathy, significant bleeding within 6 months of study entry, or urine protein: creatinine ratio >1 were excluded.

### Multiplex and single antibody immunofluorescence

The staining was performed in 10-paired TNBC biopsy samples collected before and 2 weeks after a single-dose of bevacizumab. For each patient the staining was performed on a single-biopsy pre-bevacizumab and a single-biopsy post-bevacizumab. Multiplex immunofluorescence for CD68 (Agilent Dako M0876, 1:2000), CD163 (Leica NCL-L-CD163, 1:1500), CD11c (Leica CD11C-563-L-CE, 1:1500), CD8 (Agilent Dako M710301, 1:5000), PD-1 (Cell Signaling Technology 43248 S, 1:11000), granzyme-B (Dako M7235, 1:100), CD4 (Dako M731029, 1:250), FOXP3 (BioLegend 320102, 1:2000), CD31 (Abcam Ab28364, 1:250), and Ang 2 (Santa Cruz Sc-74403, 1:250) was performed with the BOND RX fully automated autostainer (Leica Biosystems). The target antigens, antibody clones, and dilutions for all antibodies are listed in Supplementary Table 1. Formalin-fixed, paraffin embedded tissue sections were baked for 3 h at 60 °C then loaded into the BOND RX. Slides were deparaffinized (BOND DeWax Solution, Leica Biosystems) and rehydrated through a series of washes of graded ethanol to deionized water. Antigen retrieval (BOND Epitope Retrieval Solution 1, Leica Biosystems) was performed at pH 6.0 for 10 min at 98 °C. Slides were then stained with primary antibodies with an incubation time of 40 min. Next, the slides were incubated with Opal Fluorophore Reagents (Akoya Biosciences) for 5 min to visualize signal for the antibody complexes. This process was repeated iteratively for all antibodies.

Single antibody staining for CD45RO (DAKO M0742, 1:500) and MHC-I (Abcam Ab70328, 1:6000) was performed by the Dana-Farber/Harvard Cancer Center Specialized Histopathology Core. For the single CD45RA (Thermofisher MA5-12490, 1:150) antibody stain, antigen retrieval (Vector Citrate pH6 retrieval solution) was performed at pH 6.0 for 20 min at 98 °C. Slides were incubated in CuSO_4_ for 90 min to block autofluorescence. Slides were then stained with the CD45RA primary antibody overnight at 4 ^o^C followed by a Cy3 labeled anti-mouse secondary antibody (Jackson Immunoresearch). All slides were counterstained with DAPI (NucBlue Fixed Cell ReadyProbes Reagent, Invitrogen), washed with deionized water, air dried, and mounted with ProLong Diamond Anti-fade Mountant (Invitrogen).

Imaging was performed with the Mantra Quantitative Pathology Workstation (Akoya Biosciences) at 20X resolution. Images were analyzed using in QuPath^16^ and Python. Cells were identified based on a positive DAPI signal, and each of the cell populations were classified as positive or negative based on a single intensity threshold on mean expression within the cell. Immune cells located in both the tumor and stromal compartment were included in the quantitative analysis. The mean number of positive or negative cells per mm^2^ of tissue was subsequently calculated and reported.

### Stromal TIL analysis

The percentage of stromal TILs was assessed based on H&E slides using the approach reported by Salgado et al.^17^. Briefly, the tumor and stromal areas were defined and any areas with crush artifact, necrosis, or the previous core biopsy site were excluded. The type of stromal inflammatory infiltrate was determined, and all mononuclear cells (including lymphocytes and plasma cells) were included, while neutrophils and intratumoral TILs were excluded from the analysis. We analyzed one biopsy section (magnification ×200) per patient. A full assessment of average stromal TILs in the tumor area was calculated, and the area fraction of sTILs was reported.

### Statistical analysis

The Wilcoxon test was conducted for each cell population on a per patient basis for each group. An alpha value of 0.05 was considered statistically significant. Correlations were evaluated using Spearman rank correlation. Principal component analysis dimension reduction and k-means clustering with 25 random starts were conducted on all normalized patient data, pre- and post-bevacizumab. All analyses were performed using Prism Version 9 Software (GraphPad) and R Statistical Software (Foundation for Statistical Computing).

### Reporting summary

Further information on research design is available in the Nature Research Reporting Summary linked to this article.

## Supplementary information

Supplementary Information

Reporting Summary

## Data Availability

The datasets generated during and/or analyzed during the current study are available from the corresponding authors on reasonable request.

## Code availability

We generated code for QuPath 0.2.3, Python 3.7.3, and R 4.0.2. The codes used for processing of data are available from the corresponding authors on reasonable request.
