# Supplementary material for: Bevacizumab improves tumor infiltration of mature dendritic cells and effector T-cells in triple-negative breast cancer patients
Source: NPJ Precis Oncol. 2021 Jun 29;5:62. doi: 10.1038/s41698-021-00197-w (PMC8242049; doi:10.1038/s41698-021-00197-w)
Supplement: Supplementary file 1 — Supplementary Information [file 41698_2021_197_MOESM1_ESM.pdf]

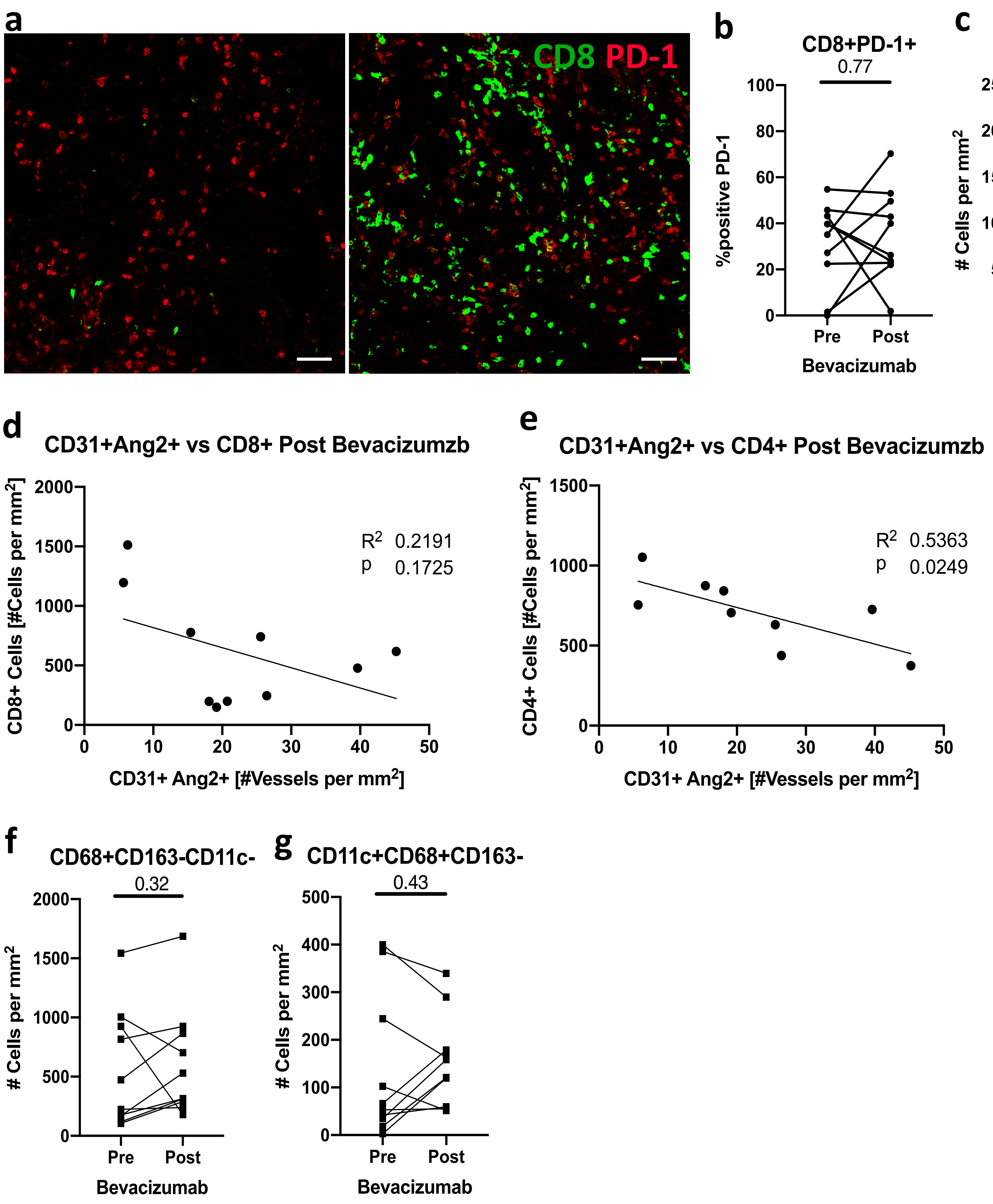

Supplementary Figure 1: Effect of bevacizumab on T cell and macrophage subsets and relationship between CD31+Ang2+ vessels and T cells. Representative images of CD8 and PD-1 immunofluorescence pre- and post-bevacizumab; Bar = 100 µm. **(b)** Fraction of CD8+PD-1+ T cells pre- versus post-bevacizumab. **(c)** Quantitative analyses of CD4+FOXP3+ cells. **(d-e)** Post- bevacizumab correlation between overall CD8+ T-cells and CD31+Ang-2+ vessels **(d)**, and CD4+ T-cells and CD31+Ang-2+ vessels **(e)**. **(f-g)** Quantitative analyses of CD68+CD163-CD11c- cells **(f)** and CD11c+CD68+CD163- cells **(g)**.

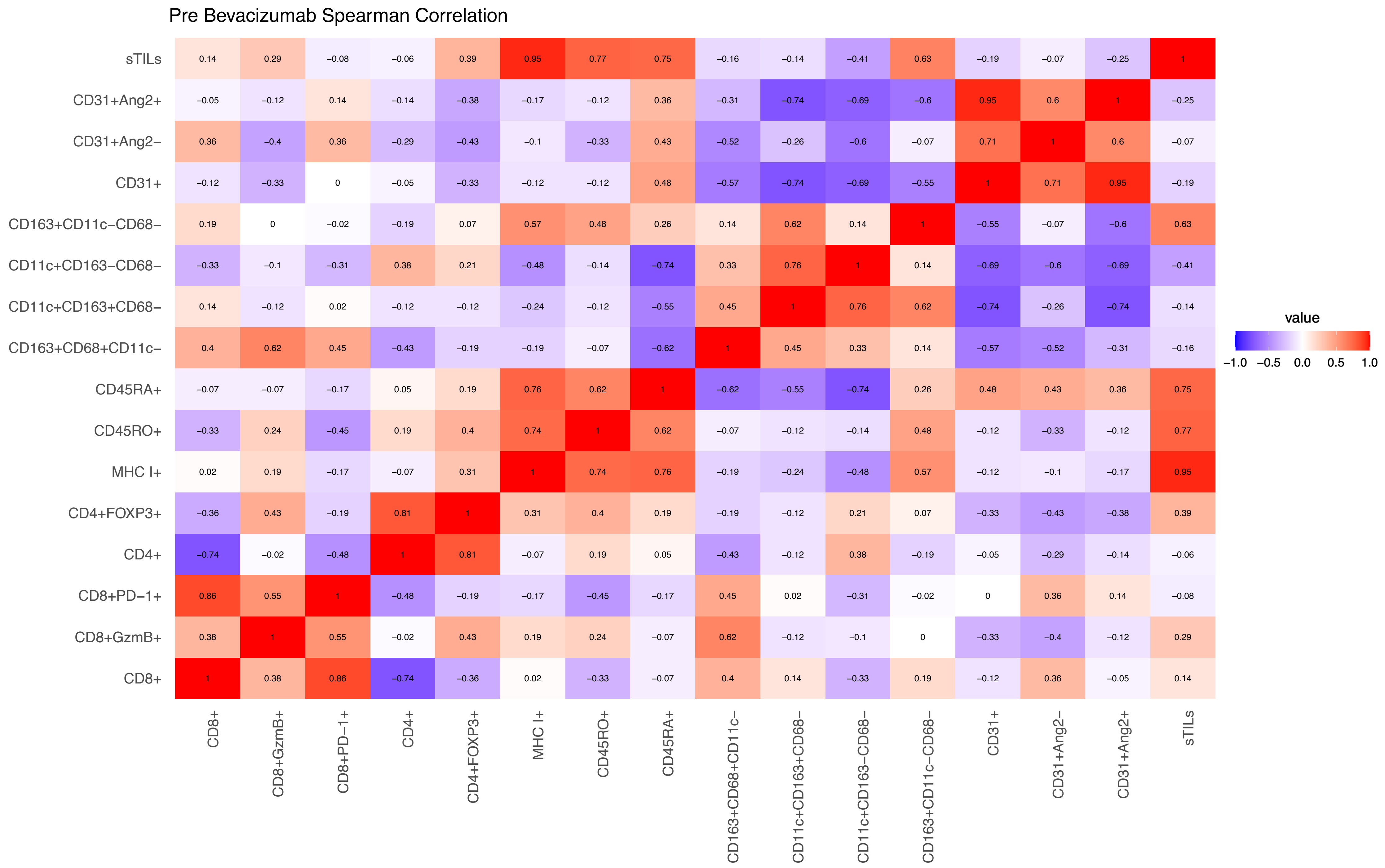

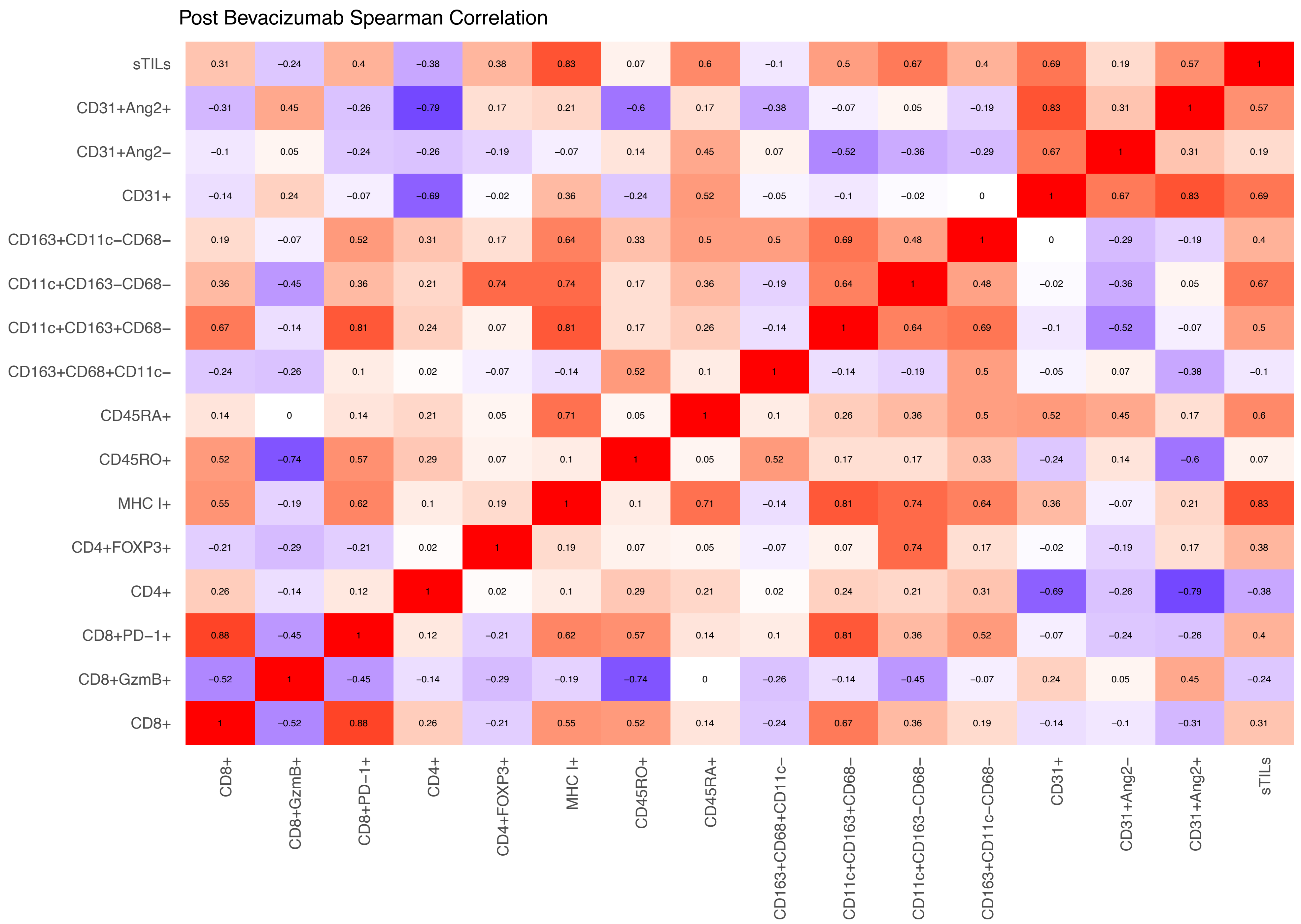

Supplementary Figure 2: Heatmap of Spearman's rank correlation of immune and vessel biomarkers pre- and post-bevacizumab. Correlation coefficients are indicated by a color scheme (legend) and discrete values between 1 and -1.

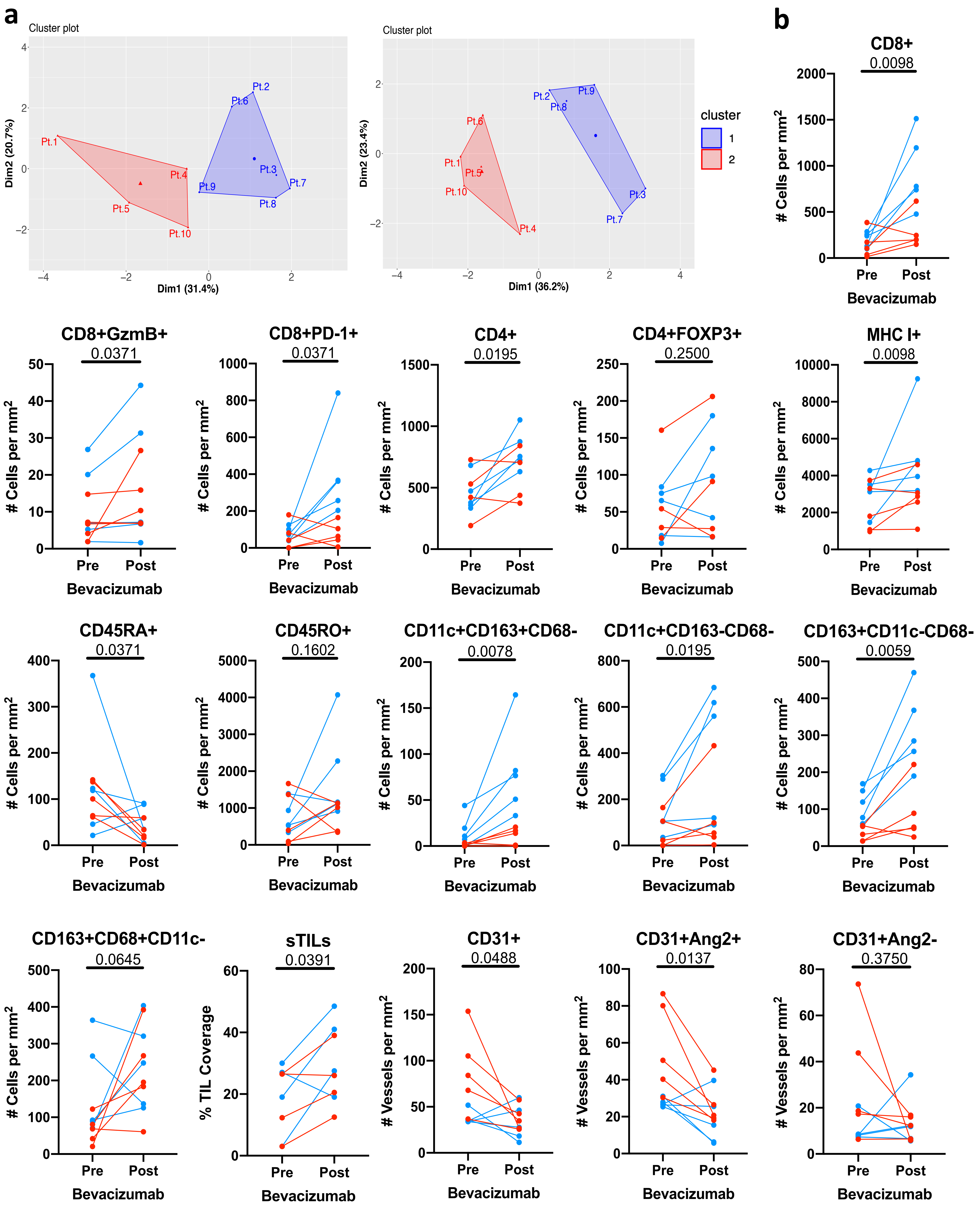

Supplementary Figure 3: Principal component analysis and k-means clustering of pre- and post-bevacizumab results. (a) Colored polygons show clustering of two patient groups pre- and post-bevacizumab. (b) Plots of 16 immune biomarkers separated in cluster 1 (blue) and cluster 2 (red) based on post-bevacizumab clustering.

Supplementary Table 1

Marker	Antibody clone	Company	Catalog #	Antibody dilution	Channel
CD68	PGM1	Agilent Dako	M0876	1:2000	540
CD163	10D6	Leica	NCL-L-CD163	1:1500	620
CD11c	5D11	Leica	CD11C-563-L-CE	1:1500	690
CD8	C8/144B	Agilent Dako	M710301	1:5000	540
PD-1	EH33	Cell Signaling Technology	43248S	1:11000	620
CD31	Polyclonal	Abcam	Ab28364	1:250	690
Ang2	F-1	Santa Cruz	Sc-74403	1:250	520
CD4	4B12	Dako	M731029	1:250	540
FOXP3	206D	BioLegend	320102	1:2000	570
Granzyme B	GrB7	Dako	M7235	1:100	620
CD45RA	4KB5	Thermofisher	MA5-12490	1:150	Cy3
CD45RO	UCHL1	Dako	M0742	1:500	AF488
MHC-I	EMR8-5	Abcam	Ab70328	1:6000	AF488
