# Supplementary material for: Bevacizumab improves tumor infiltration of mature dendritic cells and effector T-cells in triple-negative breast cancer patients
Source: NPJ Precis Oncol. 2021 Jun 29;5:62. doi: 10.1038/s41698-021-00197-w (PMC8242049; doi:10.1038/s41698-021-00197-w)
Supplement: Supplementary file 2 — Reporting Summary [file 41698_2021_197_MOESM2_ESM.pdf]

nature research

Corresponding author(s): Yves Boucher, Rakesh K. Jain

Last updated by author(s): 2021/04/26

Reporting Summary

Nature Research wishes to improve the reproducibility of the work that we publish. This form provides structure for consistency and transparency in reporting. For further information on Nature Research policies, see our Editorial Policies and the Editorial Policy Checklist.

Statistics

For all statistical analyses, confirm that the following items are present in the figure legend, table legend, main text, or Methods section.

n/a	Confirmed	
☐	☒	The exact sample size (n) for each experimental group/condition, given as a discrete number and unit of measurement
☐	☒	A statement on whether measurements were taken from distinct samples or whether the same sample was measured repeatedly
☐	☒	The statistical test(s) used AND whether they are one- or two-sided<br>*Only common tests should be described solely by name; describe more complex techniques in the Methods section.*
☒	☐	A description of all covariates tested
☒	☐	A description of any assumptions or corrections, such as tests of normality and adjustment for multiple comparisons
☒	☐	A full description of the statistical parameters including central tendency (e.g. means) or other basic estimates (e.g. regression coefficient) AND variation (e.g. standard deviation) or associated estimates of uncertainty (e.g. confidence intervals)
☒	☐	For null hypothesis testing, the test statistic (e.g. F, t, r) with confidence intervals, effect sizes, degrees of freedom and P value noted<br>*Give P values as exact values whenever suitable.*
☒	☐	For Bayesian analysis, information on the choice of priors and Markov chain Monte Carlo settings
☒	☐	For hierarchical and complex designs, identification of the appropriate level for tests and full reporting of outcomes
☒	☐	Estimates of effect sizes (e.g. Cohen's d, Pearson's r), indicating how they were calculated

Our web collection on statistics for biologists contains articles on many of the points above.

Software and code

Policy information about availability of computer code

Data collection	Mantra Quantitative Pathology Workstation
Data analysis	QuPath: 0.2.3, Python: 3.7.3, R 4.0.2

For manuscripts utilizing custom algorithms or software that are central to the research but not yet described in published literature, software must be made available to editors and reviewers. We strongly encourage code deposition in a community repository (e.g. GitHub). See the Nature Research guidelines for submitting code & software for further information.

Data

Policy information about availability of data

All manuscripts must include a data availability statement. This statement should provide the following information, where applicable:

- Accession codes, unique identifiers, or web links for publicly available datasets
- A list of figures that have associated raw data
- A description of any restrictions on data availability

The datasets generated during and/or analyzed during the current study are available from the corresponding authors on reasonable request.

Field-specific reporting

Please select the one below that is the best fit for your research. If you are not sure, read the appropriate sections before making your selection.

☒ Life sciences ☐ Behavioural & social sciences ☐ Ecological, evolutionary & environmental sciences

For a reference copy of the document with all sections, see nature.com/documents/nr-reporting-summary-flat.pdf

Life sciences study design

All studies must disclose on these points even when the disclosure is negative.

Sample size	10 paired triple-negative breast cancer biopsies (obtained before and 2 weeks after the injection of bevacizumab) were included in this study. No sample size calculation was performed. In a previous study we showed that 7 paired human triple-negative breast cancer samples was sufficient to find significant differences in vascular parameters between pre- and post-bevacizumab samples (Tolaney et al., PNAS 2015).
Data exclusions	For the analysis of stromal tumor-infiltrating lymphocytes 2/10 paired-biopsy samples were excluded because there was insufficient stroma in those samples.
Replication	No specific study has been performed yet to reproduce the specific effects of bevacizumab on the recruitment of immune cells in triple-negative breast cancer. However, in other human tumor types bevacizumab also increased the recruitment of T cells and the expression of MHC-I.
Randomization	This study was not randomized.
Blinding	The scientists who performed the imaging and quantitative analysis of immune cells and blood vessels were blinded.

Reporting for specific materials, systems and methods

We require information from authors about some types of materials, experimental systems and methods used in many studies. Here, indicate whether each material, system or method listed is relevant to your study. If you are not sure if a list item applies to your research, read the appropriate section before selecting a response.

Materials & experimental systems

n/a	Involved in the study	
☐	☒	Antibodies
☒	☐	Eukaryotic cell lines
☒	☐	Palaeontology and archaeology
☒	☐	Animals and other organisms
☐	☒	Human research participants
☐	☒	Clinical data
☒	☐	Dual use research of concern

Methods

n/a	Involved in the study	
☒	☐	ChIP-seq
☒	☐	Flow cytometry
☒	☐	MRI-based neuroimaging

Antibodies

Antibodies used

Antibody	Clone	Company	Catalogue #
CD68	PGM1	Agilent Dako	M0876
CD163	10D6	Leica	NCL-L-CD163
CD11c	5D11	Leica	CD11C-563-L-CE
CD8	C8/144B	Agilent Dako	M710301
PD-1	EH-33	Cell Signaling Tech.	43248S
CD31	Polyclonal	Abcam	AB28364
Ang2	F-1	Santa Cruz	SC-74403
CD4	4B12	Dako	M731029
FOXP3	206D	BioLegend	320102
CD45RA	4KB5	Thermofisher	MA5-12490
CD45RO	UCHL1	Dako	M0742
MHC-I	EMR8-5	Abcam	AB70328
Granzy-me B	GrB7	Dako	M7235

Validation

CD68 Agilent Daki website: monoclonal mouse anti-human CD68, immunohistochemistry

Validation	CD163 Leica website: monoclonal mouse anti-human CD163, immunohistochemistry<br>CD11c Leica website: monoclonal mouse anti-human CD11c, immunohistochemistry<br>CD8 Agilent Dako website: monoclonal mouse anti-human CD8, immunohistochemistry<br>PD-1 Cell Signaling Technology website: monoclonal mouse anti-human PD-1, immunohistochemistry<br>CD31 Abcam website: polyclonal rabbit anti-human CD31, immunohistochemistry<br>Ang2 Santa Cruz website: monoclonal mouse anti-human Ang2, immunohistochemistry<br>FOXP3 BioLegend website: monoclonal mouse anti-human FOXP3, immunohistochemistry<br>CD45RA Thermofisher website: monoclonal mouse anti-human CD45RA, immunohistochemistry<br>CD45RO Dako website: monoclonal mouse anti-human CD45RO, immunohistochemistry<br>MHC-I Abcam website: monoclonal mouse anti-human MHC-I, immunohistochemistry<br>Granzyme B Dako website: monoclonal mouse anti-human granzyme B, immunohistochemistry

Human research participants

Policy information about studies involving human research participants

Population characteristics	Enrollment required a pathological diagnosis of adenocarcinoma of the breast. Eligible TNBC patients were negative for ER, PR, and HER2, had a breast lesion ≥ 1.5 cm, and no evidence of distal metastasis. Patients with bilateral cancers were eligible as long as one cancer was eligible. Patients also required sufficient hematopoietic, hepatic, and renal function, along with a left ventricular ejection fraction ≥ 50%. Patients with any HER2-positive disease (amplified by FISH or IHC), a history of prior myocardial infarction, uncontrolled hypertension, ≥ grade 2 neuropathy, significant bleeding within 6 months of study entry, or urine protein: creatinine ratio > 1 were excluded.
Recruitment	Enrollment in this phase II trial required a pathological diagnosis of adenocarcinoma of the breast. Eligible TNBC patients were negative for ER, PR, and HER2, had a breast lesion ≥ 1.5 cm, and no evidence of distal metastasis.
Ethics oversight	This study was approved by the Dana–Farber/Harvard Cancer Center Institutional Review Board.

Note that full information on the approval of the study protocol must also be provided in the manuscript.

Clinical data

Policy information about clinical studies

All manuscripts should comply with the ICMJE guidelines for publication of clinical research and a completed CONSORT checklist must be included with all submissions.

Clinical trial registration	NCT00546156
Study protocol	https://clinicaltrials.gov/ct2/show/record/NCT00546156
Data collection	The core biopsies used in the current study were obtained in the Dept of Radiation Oncology at Dana-Farber Cancer Institute and Massachusetts General Hospital between 2007 and 2011. The multiplex immunofluorescence and quantitative analysis were performed between March 2017 and January 2021.
Outcomes	The primary and secondary outcomes of the study were not correlated with the immune cell and blood vessel data of the current article.
